# Nanomedicine of Plant Origin for the Treatment of Metabolic Disorders

**DOI:** 10.3389/fbioe.2021.811917

**Published:** 2022-02-11

**Authors:** Fang Hu, Dong-Sheng Sun, Kai-Li Wang, Dan-Ying Shang

**Affiliations:** ^1^ Medical Department, Chun’an First People’s Hospital (Zhejiang Provincial People’s Hospital Chun’an Branch), Hangzhou, China; ^2^ Department of Geriatric Medicine, Zhejiang Provincial People’s Hospital, Affiliated People’s Hospital of Hangzhou Medical College, Hangzhou, China; ^3^ Department of Cardiology, Chun’an First People’s Hospital (Zhejiang Provincial People’s Hospital Chun’an Branch), Hangzhou, China; ^4^ Department of Dermatology, Zhejiang Provincial People’s Hospital, Affiliated People’s Hospital of Hangzhou Medical College, Hangzhou, China

**Keywords:** metabolic disorders, medicinal plants, phytochemicals, nano drug, nano system

## Abstract

Metabolic disorders are major clinical challenges of health that are progressing globally. A concurrence of metabolic disorders such as obesity, insulin resistance, atherogenic dyslipidemia, and systematic hypertension leads to metabolic syndrome. Over the past years, the metabolic syndrome leads to a five- and two-fold rise in diabetes mellitus type II and cardiovascular diseases. Natural products specifically plant extracts have insulin-sensitizing, anti-inflammatory, and antioxidant properties and are also considered as an alternative option due to few adverse effects. Nanotechnology is one of the promising strategies, which improves the effectiveness of treatment and limits side effects. This review mainly focuses on plant extract-based nanosystems in the management of the metabolic syndrome. Numerous nano-drug delivery systems, i.e., liposomes, hydrogel nanocomposites, nanoemulsions, micelles, solid lipid, and core–shell nanoparticles, have been designed using plant extracts. It has been found that most of the nano-formulations successfully reduced oxidative stress, insulin resistance, chronic inflammation, and lipid profile in *in vitro* and *in vivo* studies as plant extracts interfere with the pathways of metabolic syndrome. Thus, these novel plant-based nanosystems could act as a promising candidate for clinical applications.

## Introduction

Metabolic disorders happen when the catabolic or anabolic processes are dysfunctional and incline the body to make either excessive or depleted amounts of the essential products, which are needed for the proper functioning of the body. Metabolic disorders have become a worldwide threat as they account for 20%–30% of the world’s population (Zimmet et al., 2001). Metabolic disorders are categorized into two groups: one affects the breakdown of amino acids, carbohydrates, or lipids while the other group affects the portions of the cells that produce energy. These metabolic conditions clustered together ultimately result in metabolic syndrome. Metabolic syndrome elevates the risk of several disorders comprising atherogenic dyslipidemia, cardiovascular diseases, central obesity, insulin resistance (diabetes types I and II), hypertension, and cerebrovascular accident (Tabatabaei-Malazy et al., 2015; Heindel et al., 2017). Metabolic syndrome is associated with enhanced atherosclerosis, insulin resistance, and obesity which occur due to chronic inflammation and endothelial dysfunction and also increase the risks of cardiovascular diseases and formation of abnormal adipocytokines including pro-inflammatory mediators interleukin-1 and 6 (IL-1, IL-6), tumor necrosis factor α (TNF-α), adiponectin, and leptin (McCracken et al., 2018). The World Health Organization, International Diabetes Federation, and National Cholesterol Education Program’s Adult Treatment Panel III focused on prognosis guidelines of metabolic syndrome, i.e., blood pressure values of systolic 130 mmHg or higher and diastolic 85 mmHg or higher, triglycerides 150 mg/dl or greater, high-density lipoprotein <50 mg/dl in females and <40 mg/dl in males, and elevated fasting glucose of 100 mg/dl or greater (Eckel et al., 2005).

**FIGURE 1:**
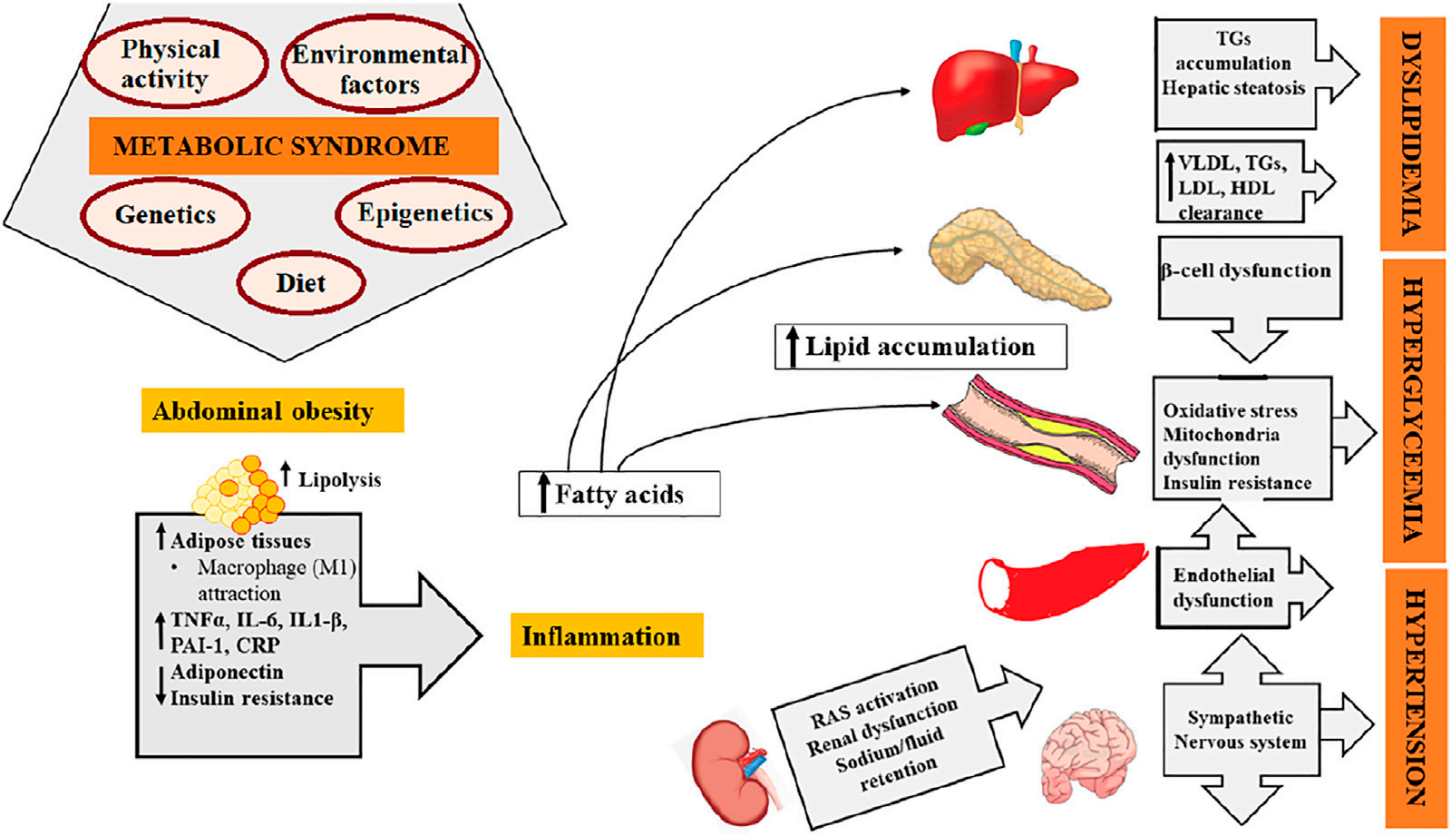
Overview of metabolic syndrome.

Several presumed mechanisms are underlying the pathophysiology of metabolic syndrome, and the most justifiable mechanism is insulin resistance with fatty acid flux. Other potential mechanisms include chronic inflammation and oxidative stress (Reaven, 1988; Roberts et al., 2013; Pan and Kong, 2018). The non-clearance of free fatty acid from circulation causes insulin resistance in obese individuals. To overcome this resistance, the pancreas secretes a large amount of insulin which results in hyperinsulinemia (Oh et al., 2018). The increased free fatty acid lessens the glucose uptake in muscle; it also causes the induction and suppression of protein kinase in the liver and muscles, respectively, ultimately leading to the elevation of gluconeogenesis (Rochlani et al., 2017). Chronic inflammation involves obesity and elevates insulin resistance, which gives rise to an abnormal production adipocytokines such as leptin, tumor necrosis factor α, prothrombotic mediator plasminogen activator inhibitor-1 (Pal-1), interleukin-1, and interleukin-6 (Vaziri et al., 2005; Di Lorenzo et al., 2013). The oxidative stress initiates insulin resistance and abolishes adiponectin production by adipocytes (Furukawa et al., 2004). Adiponectin is an essential anti-inflammatory and anti-atherogenic adipokine and acts as a protective factor against the spreading of severe diseases associated with metabolic disorders and oxidative stress, i.e., diabetes, cardiovascular diseases, and hypertension (Guerre-Millo, 2008; Becic et al., 2018). Adipose tissue stimulates the mineralocorticoid release from adrenal cells and boosts the activity of the renin–angiotensin–aldosterone system. Advancement in sodium retention and vascular tone and at the same time obstruction of norepinephrine reuptake occur which eventually lead to hypertension. Hence, it shows a parallel relationship between obesity and the pathogenesis of hypertension (Cabandugama et al., 2017). An overview of metabolic syndrome is summarized in Figure 1.

## Nano Drug Delivery Systems

Nanotechnology is a promising phenomenon to treat some diseases as it increases the bioavailability, bio-distribution, stability, and solubility of natural compounds. Numerous nano-formulations have been developed by using natural products such as liposomes, core–shell nanoparticles, hydrogels, nano-capsules, nanostructured lipid carriers, nano-emulsions, solid lipid nanoparticles, and micelles. Nano-emulsions are a suitable colloidal system for the controlled delivery of lipophilic molecules (Aswathanarayan and Vittal, 2019). Solid lipid nanoparticles can be modified through different biocompatible and biodegradable solid lipids (Ghasemiyeh and Mohammadi-Samani, 2018). Nanostructured lipid carriers are nano-carriers composed of both solid and liquid lipids (Madane and Mahajan, 2016). Nano-liposomes proved to be the best strategy to deliver and target both hydrophilic and lipophilic constituents (Khorasani et al., 2018). Core–shell nanoparticles also attain attention due to the increase in dispersibility and stability and better conjugation with bioactive compounds (Sounderya and Zhang, 2008).

The conventionally prescribed treatment for metabolic syndrome is the usual administration of drugs to lower blood glucose, triglycerides, and blood pressure. However, the long-term use of traditional drugs has a drastic side effect such as flatulence and also exerts weak tolerance for the affected person (Kaur, 2014). Consequently, nano-formulations composed of natural products have been designed to improve the efficacy and delivery of these medications (Grundy, 2016). Many studies suggested that there are several extracts isolated from medicinal plants which have anti-inflammatory, antioxidant, and insulin-sensitizing properties (Naseri et al., 2018).

### Medical Plant Extract: Polyphenols

**FIGURE 2:**
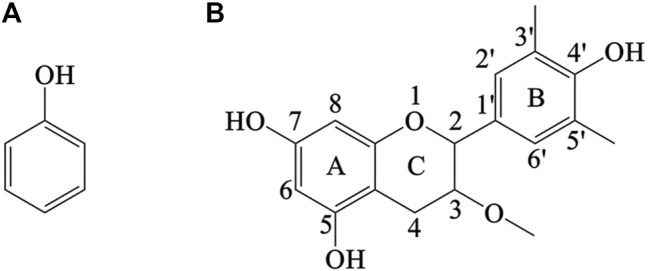
Structure of non-flavinoid **(A)** and flavinoid **(B)**.

Polyphenols or polyhydroxyphenols are the secondary metabolites of plants that contain several aromatic rings in their structure. They are famously known for their potential health benefits. They exhibit antioxidant, antibacterial, anticancer, and hypoglycemic properties. In addition, they protect against hypertension and cardiovascular diseases (Faridi Esfanjani and Jafari, 2016). Polyphenols are generally classified into two major classes, i.e., flavonoids and non-flavonoids, based on their structures. Flavonoids generally have a 15-carbon skeleton (C_6_C_3_C_6_ skeleton of carbon) composed of two phenol rings and are connected *via* an oxygen-containing central pyran ring while the non-flavonoids are composed of one phenol ring (Rambaran, 2020) (Figure 2).

### Nanoencapsulation of Polyphenols

Owing to their tremendous benefits, polyphenols have been extensively studied and various strategies have been devised to limit their side effects and optimize their efficacy. Nanoencapsulation is a potential technology to evade the barriers of poor solubility, less bioavailability and stability, and toxicity of polyphenols (Rambaran, 2020). A flavanol-myricitrin (myricetin-3-O-α-rhamnoside) is known for its antioxidant properties. Ahangarpour encapsulated the myricitrin into solid lipid nanoparticles *via* a cold homogenization method. Both *in vivo* and *in vitro* studies (streptozotocin–nicotinamide-induced diabetes mellitus type II mouse and hyperglycemic myotube) demonstrated that myricitrin solid lipid nanoparticles were effective for diabetes and hyperglycemia (Ahangarpour et al., 2018).

Ferulic acid is a polyphenol (phenolic compound) that enhances the diabetic wound healing process due to its antidiabetic, hypoglycemic, angiogenic, and free radical scavenging properties. However, lower bioavailability and less solubility in an aqueous solution hinder its use as an efficient therapeutic drug. Bairaqi synthesized ferulic acid-encapsulated nanoparticles of poly lactic co-glycolic acid through the nanoprecipitation method. Carbopol 980 hydrogel loaded with ferulic acid and poly lactic-co-glycolic acid was formulated for topical application on diabetic wounds. Both were found to be effective than the control groups and exhibited a faster epithelialization process, thus improving the diabetic wound healing process (Bairagi et al., 2018). Another bioflavonoid (flavanone), naringenin has antioxidant and antihyperglycemic properties. However, it is poorly soluble in water and has a low retention time in the intestine. Maity prepared stable therapeutic formulations of naringenin by encapsulating it in core–shell nanoparticles of chitosan and alginate. Naringenin exhibited sustained release and effective antihyperglycemic effects in rat models (Maity et al., 2017). Baicalin is a flavonoid extracted from Scutellaria radix. It demonstrates anti-hyperglycemic effects by inhibiting lipid peroxidation. Due to its less hydrophilicity and poor adsorption, it is favorable to encapsulate baicalin in nanoparticles. To encapsulate baicalin, Shah utilized nanostructured lipid carriers. The results revealed efficient retention of the drug and good stability of baicalin (Xu X et al., 2016).

Recent studies also explain how medicinal plants increase insulin sensitivity and cardiovascular function and moreover decrease gluconeogenesis, inflammation, and oxidative stress (Payab et al., 2020). The formulation of these bioactive compounds in a nanostructured form which ensured the improved bioavailability, stability, and bio-distribution of natural products (Gera et al., 2017). Plant-based nano-structured formulations have an exceptional future to treat metabolic syndrome, but there is no comprehensive review to discuss these formulations. Herein, we provide a detailed review of herbal extracted nano-formulations for metabolic disorders.

## Plant Extracted Nanosystems for Metabolic Disorders

Nanosystems are administered in the body to increase the targeting efficiency, stability, and efficacy of drugs (Kesharwani et al., 2018). However, some conventional nano-carriers have challenges such as low bioavailability, decreased efficacy, lack of targeted delivery, and high dosage (Subramani et al., 2012). Recently, an *in vitro* and *in vivo* investigation revealed that nano-systems increase the site-specific target delivery of drugs (Ponnappan and Chugh, 2015). In the case of diabetes and diabetes-related complications, biotin-fabricated nano-liposomes were found to be effective for the oral administration of insulin without leakage and also facilitated the uptake of insulin *via* receptor-mediated endocytosis (Zhang et al., 2014). Nano-formulations of conventional drugs increase the efficacy of drugs. Similarly, plant-derived nano-systems which have increased the delivery of phytochemicals and extracts for metabolic disorders are discussed as follows.

### Diabetes

Diabetes mellitus results from defects in either insulin secretion or action or both. Chronic diabetes mellitus is related to age and malfunction of various organs: kidneys, blood vessels, heart, and especially eyes. Symptoms of abnormal insulin secretion include weight loss, polydipsia, and blurred vision. Diabetes mellitus is classified into two categories based on glucose regulation: diabetes mellitus type I (β-cell dysfunction eventually leading to insulin deficiency) and diabetes mellitus type II (non-insulin-dependent diabetes or insulin resistance diabetes) (Salim, 2005). Based on published data, this review focuses on the promising role of herbal medicine incorporated in nanoparticles using nano-biotechnology as an alternative way of treating numerous metabolic disorders (Kopelman, 2000). Thymoquinone is a major bioactive component found in the seed of *Nigella sativa* (Lamiaceae family) which exhibits protection against diabetes, coronary artery disease, hypertension, oxidative diseases, and respiratory diseases (Farkhondeh et al., 2017). In a recent study, polymeric nanocapsules of thymoquinone and metformin were developed by using a biocompatible polymer gum rosin through the nanoprecipitation method and compared it with pure bioactive metformin and thymoquinone. Different dosages of polymeric nanocapsules and pure metformin were administered to diabetic rats for 21 successive days. The polymeric nanocapsules were stable, spherical, and <100 nm in diameter, and thymoquinone also gave a sustained release as compared to pure forms of metformin and thymoquinone. The oral administration of nanocapsules revealed low blood glucose levels and glycolated hemoglobin and further improved the lipid profile. Moreover, the thymoquinone-loaded nanocapsules induced a comparable anti-hyperglycemic effect against metformin nanocapsules, pure thymoquinone, and metformin (Rani et al., 2018). The feature of insulin resistance in diabetes mellitus type II accounts for 90%–95% of the diabetic population which fails insulin function. Recently, Zhang synthesized self-assembled micelles *via* the conjugation of plant-derived polymer polygalacturonic acid (PGA) and natural insulin sensitizer oleanolic acid (OA) which exhibited as an oral nano-carrier for the treatment of diabetes mellitus type II. The *in vitro* and *in vivo* studies revealed that OA-loaded PGA-OA micelles improved the permeability of the gastrointestinal barrier, enhanced the drug intestinal absorption, and maintained the plasma drug concentration for a longer period. Furthermore, the administration of nano-formulation in diabetes mellitus type II model rats demonstrated a tremendous effect on insulin resistance and controlled the blood glucose level and gave a long-termed effect even after its withdrawal (Zhang et al., 2020).

*Pouteria sapota* is commonly found in Mexico and South America which has a significantly higher antioxidant activity due to the presence of polyphenols (Ma et al., 2004). The green synthesis method was used to prepare silver nanoparticles using the extract of *P. sapota*, and their antidiabetic activity was evaluated in cellular and animal models. The *in vitro* antidiabetic activity of silver nanoparticles was confirmed due to the decrease in non-enzymatic glycosylation, hindrance of α-amylase, and increase in glucose uptake by yeast cells. On the other hand, the biosynthesized silver nanoparticles significantly improved the superoxide dismutase and catalase activity, enhanced the plasma insulin level, and decreased the blood glucose level in streptozotocin-induced rats (Prabhu et al., 2018). Betalain is a natural constitute of cactus pear (*Opuntia* spp.), red beetroot (*Beta vulgaris*), pitahayas (*Stenocereus* spp.), and amaranth (*Amaranthaceae*) (Sawicki et al., 2018), which displays antidiabetes (Dhananjayan et al., 2017), anti-carcinogenic (Amjadi et al., 2019a), and anti-inflammatory properties (Tan et al., 2015). However, poor oral absorption and stability limit its application. To overcome these challenges, Amjadi synthesized betalain-loaded nano-liposomes. A sustained *in vitro* release profile was observed in both simulated gastric and intestinal fluid. *In vivo* administration of nano-carriers in streptozotocin-induced rats demonstrated improved regulation of hyperglycemia, oxidative stress, and hyperlipidemia as compared to free betalain. Additionally, the histopathological analysis of diabetic rats revealed reduced tissue in the pancreas, liver, and kidney (Amjadi et al., 2019b).

Selenium is an important trace element in a body that protects the immune system and maintains homeostasis. When selenium is incorporated into nanoparticles, it gives a novel nutritional supplementation with lowered toxicity due to the sustained delivery after its ingestion (Skalickova et al., 2017). Previously, selenium-layered nanoparticles (SeNPs) were synthesized for the delivery of mulberry leaf and *Pueraria lobata* extracts (MPE) which have hypoglycemia properties and give an antidiabetic effect. The MPE-loaded selenium nanoparticles (MPE-SeNPs) were prepared *via* solvent diffusion/*in-situ* reduction method. The MPE-SeNPs were 120 nm in diameter with 89.38% and 90.59% encapsulation efficiency for rutin and puerarin, respectively. The MPE-SeNPs gave a controlled release and improved stability in simulated digestive fluid. The oral administration of MPE-loaded selenium nanoparticles produced a remarkable hypoglycemic effect in control and diabetic rat. An outstanding intestinal permeability was observed in *ex vivo* intestinal examination (Deng et al., 2019). One of the major causes of diabetes mellitus is oxidative stress; myricitrin is an antioxidant derived from plants, and its solid lipid nanoparticles are much in demand to enhance the antidiabetic and antiapoptotic effects. Myricitrin solid lipid nanoparticles were prepared through the cold homogenization method, and its effects on streptozotocin–nicotinamide-induced diabetes mellitus type II mouse and hyperglycemic myotube were observed. Myricitrin solid lipid nanoparticles demonstrated improved hyperglycemic and diabetes complications (Ahangarpour et al., 2018). Naringenin is a member of the flavonoid family which is found in vegetables and citrus fruits (grapefruits and oranges) (Cavia-Saiz et al., 2010). It has shown anti-hyperglycemic, anticancer (Pateliya et al., 2021), antioxidant (Yin et al., 2020), and anti-inflammatory (Gratieri et al., 2020) properties. The major limitations of naringenin are its poor solubility in water and lower bioavailability due to its metabolism by gut and liver enzymes (Manach et al., 2004). Core–shell nanoparticles of alginate/chitosan loaded with naringenin were prepared through Na_2_SO_4_ and CaCl_2_ cross-linking to overcome the limitations of naringenin. The developed nanoparticles were characterized using different techniques. The encapsulation and amorphous nature of naringenin-loaded core–shell nanoparticles were confirmed by Fourier-transform infrared spectroscopy and x-ray diffractogram, respectively. The average hydrodynamic size of nanoparticles ranges between 150 and 300 nm with spherical and smooth morphology. The release kinetics of naringenin showed 15% release in simulated gastric fluid (pH = 1.2) while >90% of the drug was released in a sustained manner *via* simulated intestinal fluid (pH = 7.4). The *in vivo* study revealed streptozotocin-induced mice reduced the blood glucose levels and enhanced the hypoglycemic effect after oral administration of nanoparticles due to stimulatory activity of naringenin. Furthermore, the histopathological and blood analysis indicated a non-toxicity of nano-system (Maity et al., 2017). Some other nanosystems which are providing effective treatment of diabetes in mice are mentioned in Table 1.

**TABLE 1:** Plant extract-based nanosystems for treatment of diabetes.

Medicinal plants	Nano-systems	Experimental model	Explanation	References
*Salvia sclarea*	Solid lipid nanoparticles	HFD-induced diabetic mice	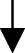 Adipocytes	Cerri et al. (2019)
			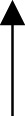 Brown adipose tissue weight; HDL level;	
			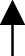 Improved glycemic level	
*Solanum nigrum*	Silver nanoparticles	Diabetic rat	Improved dyslipidemia	Sengottaiyan et al. (2016)
			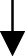 Blood glucose level	
Curcumin	Nanoparticles	Diabetic albino rat	Glucose-lowering effects; antioxidant effects; insulin level	Abu-Taweel et al. (2020)
*Catathelasma ventricosum*	Selenium nanoparticles	STZ-induced diabetic rats	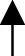 Antidiabetic effect	Liu L et al. (2018)
			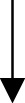 Body weight; antioxidant activity; lipid level	
Silymarin	Nanostructured lipid carriers	STZ-induced diabetic rats	Blood glucose level; triglycerides; anti-hyperalgesic effects	Piazzini et al. (2019)
Curcumin	Liposomes	STZ-induced diabetic rats	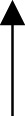 Hypoglycemic, hypoprotective, and antioxidant effects	Bulboacă et al. (2019)
			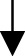 Oxidative stress	
Quercetin	Core–shell nanoparticle (chitosan/alginate)	Diabetic rat	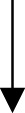 Blood glucose level	Mukhopadhyay et al. (2018)
			Hyperlipidemic activity	
*Annona muricata*	Silver nanoparticles	HaCat cell lines	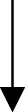 Blood glucose level	Badmus et al. (2020)
			α-Amylase and α-glucosidase activity	
*Talinum portulacifolium*	Solid lipid nanoparticles	STZ- and HFD-induced diabetic rats	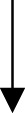 Blood glucose level; serum insulin; TGs	Bindu et al. (2014)
			Lipid profile	
Myricitrin	Solid lipid nanoparticles	STZ NA-induced diabetic rat	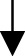 Oxidative stress	Ahangarpour et al. (2019)
			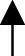 Antioxidant enzyme level	

HFD, high-feed diet; HDL, high-density lipoprotein; STZ, streptozotocin; TGs, triglycerides; NA, nicotinamide; HaCat, immortalized human keratinocytes.

### Diabetic Wound Healing

Wound healing is a normal process of a human body after any injury which is achieved through a programmed set of phases, i.e., homeostasis, inflammation, proliferation, and remodeling. These phases should occur in this proper sequence and time frame to properly heal a wound (Guo and DiPietro, 2010). Diabetic wound healing and diabetic foot ulcers are the major causes of amputations which affect 15% of the patients suffering from diabetes. The major cause of diabetic wound healing is decreased cell growth factor response which abolishes the blood flow and low local angiogenesis (Brem and Tomic-Canic, 2007). During the past few years, nanotechnology has gained attention, which allows a sustained and site-specific delivery of bioactive compounds. Various nanoformulations that are treating diabetic wounds are presented in Table 2. Quercetin (QCT) is found in various medicinal plants, i.e., *Hypericum perforatum*, *Ginkgo biloba*, and *Sambucus canadensis*, which act as a wound healing, antioxidant, anti-carcinogenic, anti-inflammatory, and anti-fibrotic agent.

**TABLE 2:** Nanoformulations for diabetic wound healing.

Medicinal plants	Nano-systems	Model animals	Explanation	References
*Saraca asoca*	Silver nanoparticles	Swiss albino mice	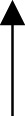 Re-epithelialization; wound contraction	Bairagi and Nath (2021)
*Bambusa bambos*	Nanobiocomposite	STZ-induced diabetic rats	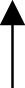 Re-epithelialization;	Singla et al. (2017a)
			Collagen deposition	
*Dendrocalamus hamiltoni*	Nanobiocomposite	STZ-induced diabetic rats	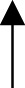 Re-epithelialization;	Singla et al. (2017a)
			Collagen deposition	
Curcumin	Chitosan nanoparticles	STZ-induced diabetic rats	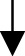 Macrophage-induced	Li et al. (2019)
			Inflammation	
			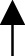 Angiogenesis	
Curcumin	Curcumin nanoparticle-loaded hydrogel	STZ-induced diabetic albino rats	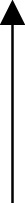 Wound closure rate; Granulation tissue formation; collagen Deposition.	Kamar et al. (2019)
*Syzygium cumini*	Nanocomposites	STZ-induced diabetic rats	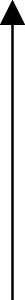 Fast re-epithelialization; neo-vascularizationCollagen deposition Anti-inflammatory Action	Singla et al. (2017b)
*Acalypha indica*	Gold nanoparticles	BALB/c mice	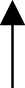 Re-epithelialization	Boomi et al. (2020)
			Collagen deposition	
*Pterocarpus marsupium*	Chitosan nanoparticle-loaded hydrogel	STZ-induced diabetic rat	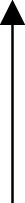 Re-epithelialization; growth of granular	Manne et al. (2021)
			Tissues; collagen	
			Deposition	

STZ, streptozotocin; BALB/c mice, albino immunodeficient inbred strain (Bragg albino).

Badhwar et al. (2021) prepared a hydrogel with quercetin-loaded silver nanoparticles (QCT-AgNPs) which are considered as a gold standard for the treatment of diabetic and burnt wounds. The optimized hydrogel demonstrated 92.09% entrapment efficiency and smooth surface morphology with a 44.1-nm hydrodynamic diameter. A relatively higher antimicrobial activity against *S. aureus and E. coli* was observed by QCT-AgNP hydrogel in comparison with marketed hydrogel. Furthermore, the *in vivo* results revealed that QCT-AgNP hydrogel decreased the wound gap and enhanced the % re-epithelialization in the diabetic wound model. Diabetic wounds have serious challenges, which may lead to amputation of the lower extremities. Recently, for the co-delivery of curcumin (CUR) and resveratrol (RES), novel hyaluronic acid (HA)–functionalized chitosan nanoparticles (HA-CUR-RES-CS-NPs) were prepared through the ionic cross-linking method. The fabricated nanoparticles had a particle size <200 nm, zeta potential > ± 30 mV, and entrapment efficiency of 90%. The *in vitro* release pattern revealed a non-Fickian diffusion and sustained a release mechanism (Hussain et al., 2020).

A novel nano-hybrid scaffold was prepared by encapsulation of curcumin into chitosan nanoparticles (CUR-CS NPs) followed by its impregnation into a collagen scaffold for improved tissue regeneration. The nanoparticles improved the stability and solubility of curcumin. A sustained *in vitro* release, outstanding biocompatibility, and drug availability were found in the case of a nano-hybrid scaffold. The *in vivo* wound closure analysis also demonstrated that scaffold-treated wounds healed more efficiently as compared to control and placebo scaffolds. A formation of thick granulation tissue and complete epithelialization were observed in nano-hybrid scaffolds (Karri et al., 2016). Ponnanikajamideen and coworkers (Ponnanikajamideen et al., 2019) used the green method for the plant-mediated synthesis of gold nanoparticles by using the extract of *Chamaecostus cuspidatus* (insulin plant) to study the hypoglycemic effect in healthy rats and streptozotocin-induced diabetic rats. The green synthesized gold nanoparticles were evaluated through transmission electron microscopy, scanning electron microscopy, and free-radical scavenging activity. The characterized nanoparticles showed spherical morphology with 20 nm of size. The antidiabetes studies revealed that the extract had a significant hypoglycemic effect as compared to control groups. The free radicals were exhibited in a dose-dependent manner, and 50% inhibition of free radicals was observed by treating with gold nanoparticles. Moreover, the cutaneous wound healing activity of nanoparticles gave comparable wound recovery in comparison with controls. The toxicity analysis in mice showed controlled blood glucose, glycogen, and serum levels.

Another nano-system is designed to heal diabetic wounds efficiently and increase the availability of curcumin. Curcumin is a polyphenolic compound that has therapeutic effects, but low bioavailability and *in-vivo* stability have limited its use (Manju and Sreenivasan, 2011). However, the delivery of curcumin through a carrier enhanced the release and also increases its bioavailability. A thermo-sensitive hydrogel in a structure of gelatin microspheres loaded with curcumin was prepared. At first, the self-assembly of curcumin nanoparticles was done followed by its encapsulation in the gelatin macrospheres to respond to matrix metalloproteinase (which are overexpressed in diabetic wounds). The hydrogel containing curcumin loaded in gelatin microspheres was delivered to wound sites to investigate the release and healing efficiency in streptozotocin-induced diabetic mice. The results revealed that this developed delivery system significantly promoted the healing process in mice which have the potential to become a skin drug delivery system (Liu Y et al., 2018).

### Obesity

Obesity is the world’s most common disease and is considered a root for many metabolic disorders. Obesity is usually associated with diabetes, cardiovascular diseases, and some forms of cancers. The parameter to define obesity is body mass index. Insulin resistance is a major consequence of obesity. There is a string associated with elevated fat cells and diabetes that makes the release of interleukin-6 from fat cells that triggers the pro-inflammatory state indicating obesity (Hill et al., 2003). To treat obesity through nano-systems, *Salacia chinensis-*loaded gold nanoparticles (SC-AuNPs) were designed to evaluate the antiobesity parameters in obese rats. After the formation of SC-AuNPs, the nanoparticles were optimized using different techniques, i.e., ultraviolet-visible spectroscopy, x-ray diffraction, Fourier-transform infrared spectroscopy, scanning electron spectroscopy, energy-dispersive x-ray analysis, and transmission electron microscopy. The treated rats were analyzed to check the change in body weight index, adiponectin, lipid profile, leptin, liver marker enzymes, resistin, inflammatory markers, AMP-activated protein kinase, and liver histo-pathophysiology. The results revealed a spherical morphology, various functional groups, and crystalline nature of SC-AuNPs. The optimized nano-formulation decreased the body weight, resistin, liver marker enzymes, leptin, adipose index, and inflammatory markers. Additionally, the SC-AuNP treatment increased the high-density lipoprotein, AMP-activated protein kinase, and adiponectin. The histopathological profile showed lower hepatocyte degradation due to the SC-AuNPs (Gao et al., 2020). In another study, Ansari and coworkers developed *Smilax glabra* rhizome-based gold nanoparticles to treat obesity in the streptozotocin-induced rat model. The prepared nanoparticles were characterized using different techniques: ultraviolet-visible spectroscopy, scanning electron spectroscopy, X-ray diffraction, Fourier-transform infrared spectroscopy, and transmission electron microscopy. The ultraviolent-visible spectrum of gold nanoparticles was recorded at the wavelength of 530 nm. The shape and diameter of the gold nanoparticles were hollow and 50–90 nm, respectively. The chemical binding and crystal form of nanoparticles were confirmed through Fourier-transform infrared spectroscopy and X-ray diffraction. Various biochemical parameters, i.e., blood glucose, insulin sufferance and its release, liver markers, lipid profile, and hormones such as adiponectin, leptin, and resistin, indicated the therapeutic effects of nanoparticles on rats. Moreover, the histopathological analysis showed that the distorted liver and cardiac tissues restored membrane, cytoplasm, and nuclei upon treatment with *Smilax glabra*-derived gold nanoparticles (Ansari et al., 2019).

Saratale prepared silver nanoparticles using the extract of *Argyreia nervosa* to evaluate *in vitro* inhibitory effects on α-amylase and α-glucosidase which are essential enzymes for carbohydrate metabolism. The prepared silver nanoparticles were spherical in shape, and the average diameter was about 15 nm. The IC_50_ values of α-glucosidase and α-amylase were 51.7 and 55.5 μg/ml, respectively, showing antidiabetic potential. The increase in the surface area and entrapment of free radicals was observed due to the attachment of functional groups of phytochemicals on AgNPs. In addition, the silver nanoparticles displayed strong antibacterial activity against *Escherichia coli* and *Staphylococcus aureus* (Saratale et al., 2017). In recent times, numerous nanosystems have been designed for obesity; a few are summarized in Table 3.

**TABLE 3:** Plant extract-derived nanosystems for obesity.

Medicinal plants	Nano-systems	Experimental models	Explanation		References
*Oleoresin capsicum*	Single-layer and alginate double-layer nano-emulsion	HFD-induced obesity in rat and 3T3-L1 cell lines	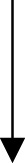	Lipid level; TGs; mRNA level of PPAR- γ; fatty	Lee et al. (2017)
				Acid-binding protein	
				Adipocyte	
*Citrus sinensis*	Nano-vesicles	HFHSD mice	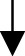	Chylomicron synthesis	Berger et al. (2020)
				TGs; plasma lipids	
			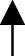	Villi size	
*Dendropanax morbifera*	Gold nanoparticles	3T3-L1 &amp; HepG2 cell lines	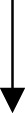	TGs; PPAR- γ; Jak2	Yi et al. (2020)
				STAT3; CEBPα; ap2 expression	

HFD, high-feed diet; TGs, triglycerides; PPAR-γ, peroxisome proliferator-activator receptor-gamma; HFHSD, high-fat, high-sucrose diet; Jak-2, Janus kinase 2 (non-receptor tyrosine kinase); STAT3, signal transducer and activation of transcription 3; CEBPα, transcription regulator and enhancer; ap2, a transcription factor expressed in adipose tissues.

The browning of white adipose tissues (WAT) *via* enhancing thermogenic energy expenditure is one of the therapeutic approaches to regulate the energy imbalance and problems associated with excess body weight. The detrimental effects of this method limited its use. To overcome the foremost issue, ligand fabricated-resveratrol-loaded nanoparticles (L-Rnano) were prepared that specifically bind to decorin receptors on adipose stromal cells. The transmission electron microscopic images of L-Rnano showed a spherical shape with a size of 90–110 nm. The intravenous administration of L-Rnano to obese C57BL/6J mice remarkably induced adipose stromal cell differentiation into beige adipocytes which ultimately reduced the 40% fat mass, inflammation, and enhanced glucose hemostasis (Zu et al., 2021).

In another study, a novel transdermal drug delivery carrier was designed to reduce the volume of subcutaneous adipose tissues. Curcumin-containing poly-vinyl alcohol gelatin nanofibers were synthesized ranging from 200 to 250 nm in diameter. A uniform method was used for the preparation of transdermal patches. The efficacy of the delivery system in the transport of curcumin through the skin is proved by adjacent arrangement transdermal diffusion cells. The transdermal patches demonstrated reduced the number of adipose tissues up to 4%–7% in model rats (Ariamoghaddam et al., 2018). Similarly, a soy extract-based topical drug delivery system was designed to determine the antiobesity action of soy topically on high-fed diet-induced mice. Nano-sized phytosomes were formulated *via* the thermogel method. The optimized formulation had encapsulation efficiency % of 99.89, and gel transformation temperature was recorded to be 31.5°C. Fourier-transform infrared spectroscopy also confirmed the soy entrapment in the nanosystem due to the formation of a hydrogen bond between OH groups of soy extract and phosphatidylcholine which eventually increased the permeation rate. The nano-phytosome formulation of soybean revealed an improved release pattern (92.50% within 2 h). Moreover, the *in vivo* study on the mouse model demonstrated that soy extract had reduced the size of adipose cells with slight lowering effects on triglycerides and low and very-low-density lipoprotein levels (El-Menshawe et al., 2018).

### Dyslipidemia

Dyslipidemia is one of the major metabolic disorders which occur due to the abnormalities in lipid profile such as higher levels of Apo B, triglycerides, very-low-density lipoprotein, and low-density lipoprotein with a decreased level of high-density lipoprotein (Sun et al., 2018). The anomalies in the structure, function, and metabolism of atherogenic and anti-atherogenic lipoproteins result in unhealthy lipid levels (Halpern et al., 2010). Malnutrition and a sedentary lifestyle are the other reasons for dyslipidemia, but a prolonged increase in insulin levels ultimately leads to atherogenic dyslipidemia in different ways. Firstly, the disruption in insulin signaling increases lipolysis which causes the production of free fatty acids and very low-density lipoproteins in hepatocytes. Secondly, the insulin involves in Apo B degradation and lipoprotein lipase activity which causes the hypertriglyceridemia to elevate very-low-density-lipoprotein formation and its storage. Triglycerides are collected from very-low-density lipoprotein/low-density lipoprotein and get exchanged for cholesteryl esters which result in triglyceride-rich high-density lipoproteins. These high-density lipoproteins are immediately cleared by hepatic lipases and removed from circulation (Srikanth and Deedwania, 2016).

Dyslipidemia is one of the risks for the progression of cardiovascular diseases such as atherosclerosis, ischemic heart disease, stroke, and coronary heart disease because it leads to the synthesis of free radicals and oxidative stress (Georges et al., 2019; Singh et al., 2016). Conventional medications for dyslipidemia include lovastatin, atorvastatin, simvastatin, and pravastatin which have adverse effects, i.e., myopathy, rhabdomyolysis, and myalgia (Shin et al., 2014; Chu et al., 2015; Wat et al., 2016). To overcome these challenges, natural products such as garlic oil and kenaf oilseed are encapsulated in nanosystems to give antioxidant and anti-hyperlipidemic effects. Few other medicinal plant-extracted nanosystems are explained in Table 4. Garlic (*Allium sativum L*.) oil consists of sulfur-containing compounds that have anti-hyperlipidemic (Keshetty et al., 2009), antimicrobial (Zheng et al., 2013), antioxidant, (Ebrahimzadeh-Bideskan et al., 2016), and antidiabetic (Sambu et al., 2015) properties. Ragavan formulated garlic oil nano-emulsion through ultrasonic emulsification, and the optimized nano-emulsion showed spherical morphology with a droplet size of 24.9 ± 1.11 nm. The zeta potential of formulated nano-emulsion was -42.63 ± 1.58 mV, and the PDI value was low. Small size, negative zeta potential, and low PDI values collectively gave stability to the nano-system. The acute toxicity study revealed that nano-emulsion of garlic oil with Tween as a surfactant did not exhibit any toxicity. Garlic oil nano-emulsion when administered to dyslipidemic Wistar rats demonstrated a significant effect in lowering the lipid profile in comparison with pure drug (atorvastatin) and garlic oil. In addition, the lipid deposits in hepatic tissues were also reduced when analyzed under Oil Red O staining which suggested that the developed nano-emulsion is a promising candidate for treating dyslipidemia (Ragavan et al., 2017). *Ziziphus jujube* (jujube) is a medicinal fruit that has antioxidant, anti-inflammatory, hepatoprotective, antibacterial, and anti-inflammation properties. The green synthesis of gold nanoparticles using jujube was done followed by its characterization based on transmission electron microscopy and X-ray diffraction. The smooth spherical morphology and 7–27-nm-sized gold nanoparticles were obtained. For *in vivo* studies, different doses of gold nanoparticles were administered to streptozotocin-induced diabetic rats, and lipid profile, body weight, insulin, and liver oxidative markers were evaluated. The results showed a significant decrease in the levels of liver, insulin, triglycerides, cholesterol, and total antioxidant capacity (Javanshir et al., 2020).

**TABLE 4:** Nanosystems for dyslipidemia.

Medicinal plants	Nano-systems	Model animals	Explanation	References
Black currant	Selenium nanoparticles	Galactose-treated rats	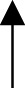 Hypolipidemia Antioxidation activity	Al-Kurdy and Khadim Khudair (2020)
*Nigella sativa*	Silver nanoparticles	Male adult rats	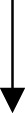 TGS; cholestrol absorption; LDL-c; oxidative stress 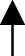 HLD-c	Ali et al. (2019)

TG, triglycerides; LDL, low-density lipoprotein; HDL, high-density lipoprotein.

Kenaf (*Hibiscus cannabinus L.*) seed oil has phytosterol and saponin (Shi et al., 2004) which reduce the cholesterol level, but the hydrophobic nature and poor bioavailability have limited its applications. Nano-emulsion and macro-emulsion of kenaf seed oil were prepared to investigate liver oxidative status, lipid serum profile, and histopathological changes in high cholesterol diet-induced rats. The kenaf seed oil in water nanoemulsion displayed narrow particle size distribution and higher zeta potential indicating the high electrostatic interaction between the particles. The stability and encapsulation and bioavailability of kenaf seed oil in water nano-emulsion were higher than macroemulsion. *In vivo* studies revealed that the nano-emulsion declined the accumulation of fat droplets in the liver, lowered cholesterol, decreased the number of endogenous antioxidants in the liver, and controlled the weight in high cholesterol diet-induced rats. Furthermore, the histopathological analysis on rats suggested an accelerated renewal of liver cells after injury was observed due to nano-emulsion (Cheong et al., 2018).

### Hypertension

Hypertension is becoming a serious threat worldwide, which refers to the rise in arterial blood pressure. An adequate amount of blood is required throughout the circulation; otherwise, it damages the eyes, kidneys, and brains and also leads to heart-related problems, i.e., cardiac failure, myocardial infarction, stroke, and peripheral vascular disease (Alam et al., 2017; Kikuchi et al., 2018). A modified approach is needed to limit cardiovascular diseases. In a study, curcumin-loaded poly (lactic-co-glycolic acid) nanoparticles were prepared by a single emulsion method to evaluate the cardiovascular parameters in high-fed diet Wistar rats. The hydrodynamic diameter and zeta potential of nanoparticles were obtained to be 315–320 nm and −29 mV, respectively. The greater size marked due to the higher molecular weight of poly (lactic-co-glycolic acid) and negative zeta potential confirmed the stability of the nano-system in circulation. The results revealed that a 20-fold lower dosage of curcumin in poly (lactic-co-glycolic acid) nanoparticles normalized the blood pressure, reduced liver fat deposition, and improved ventricular inflammation and fibrosis (du Preez et al., 2019).

High blood pressure in the arteries of the lung causes a progressive disorder known as pulmonary arterial hypertension. Inflammation, oxidative stress, and nitric oxide are involved in the development of pulmonary arterial hypertension (Xu et al., 2017). It disturbs the vascular function by increasing the vascular resistance and obstructing the pulmonary artery, which eventually leads to right ventricular hypertrophy and right-sided heart failure. The medicinal plants which have antioxidant and anti-inflammatory activities can treat pulmonary arterial hypertension (Xiang et al., 2018; Meghwani et al., 2018; Xu D et al., 2016). Copaiba oil is an oil-resin that comes from an Amazonian tree and is used as an herbal treatment in Brazil. The major composition of copaiba oil is β-caryophyllene which is a calcium channel blocker. The blocker has an antioxidant and anti-inflammatory action and also has inhibitory effects on cell growth (Rasheed et al., 2015; Ames-Sibin et al., 2018). Copaiba oil-loaded nanocapsules were prepared to investigate the monocrotaline-induced pulmonary arterial hypertension. The free copaiba oil and nanocapsules enhanced the sulfhydryl groups, superoxide dismutase, and Nrf2 (antioxidant transcription factor) and removed the oxidized glutathione concentration, but the nano encapsulated oil was more effective than free copaiba oil. Both the oil and nano-formulation significantly reduced the right ventricular hypertrophy index. However, the nanosystem did not show any effect on pulmonary vascular resistance. Furthermore, the nano-capsules composed of pectin and copaiba oil have enhanced the pharmacological effect (Campos et al., 2017). In short, the nano-encapsulation of copaiba oil provides the favorable delivery and also increased its efficiency. Some nano-formulated medicinal plants that have given different therapeutic effects are listed in Table 5.

**TABLE 5:** Plant extract-based nanosystems for treatment of hypertension.

Medicinal plants	Nano-systems	Model animals	Explanation		References
Curcumin	PLGA nanoparticles	HFD-induced mice	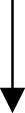	Systolic blood pressure Ventricular stiffness; Fat deposition	du Preez et al. (2019)
Curcumin	Nanoparticles	Male Sprague Dawley rats	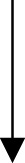	Right ventricular wall thickness; right Ventricle weight/body ratio; oxidative stress	Rice et al. (2015)
*Copaifera* sp.	Nano-capsules	Wistar rats	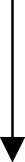	Right ventricle hypertrophy; oxidative Stress; pulmonary Vascular resistance	Campos et al. (2017)

PLGA, poly (lactic-co-glycolic acid); HFD, high-feed diet.

## Conclusion

**FIGURE 3:**
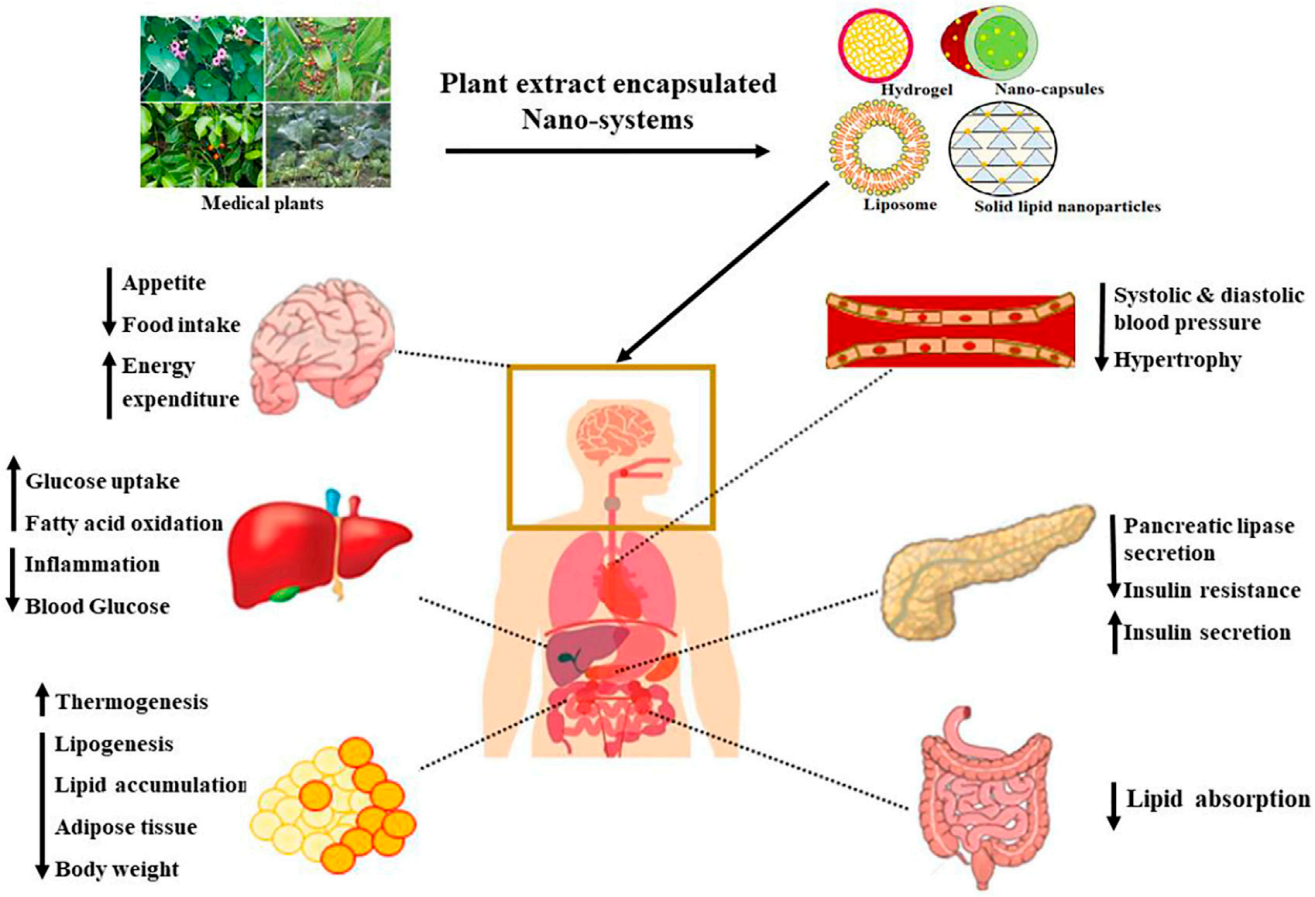
Importance of plant-extract-encapsulated nanosystems for metabolic disorders.

Metabolic syndrome is a complex disorder comprising insulin resistance, hyperinsulinemia, and impaired glucose tolerance. There is a need for an effective strategy to treat the complications in the pathophysiology of metabolic syndrome. Despite extensive research on the therapeutic effects of plant-derived bioactive compounds, their delivery and bioavailability are always troublesome. Over the past few years, the nanoencapsulation of bioactive compounds has revolutionized the pharmaceutical and clinical industries. These nanosystems have improved not only the bioavailability but also the efficacy, stability, and solubility of plant extracts. When these plant extract-encapsulated nanoformulations are administered to the body, some metabolic changes are expected to occur which are summarized in Figure 3.

Diabetes mellitus is a challenging and problematic disease; however, nano-formulations of plant extracts have shown remarkable antidiabetic effects in comparison with conventional treatments. Thymoquinone incorporated in nanocapsules, betalain within selenium nanoparticles, naringenin-encapsulated core–shell nanoparticles, and various nanosystems exhibited hypoglycemia and low lipids levels, hence providing effective alternative therapeutics for diabetes. Similarly, the anti-inflammatory and antibacterial properties of plant extract-based nanosystems have provided tremendous advantages in rapid wound healing. Among all the studies in this review, thermosensitive hydrogel loaded with curcumin nanoparticles showed a fast recovery in streptozotocin-induced mice. The delivery of plant extract *via* chitosan/alginate core–shell nanoparticles induced a significant hypoglycemic effect and decreased oxidative stress in antiobesity therapy. The nanoemulsions of sulfur-containing medicinal plants such as *Allium sativum L.* and kenaf seed oil showed the highest anti-hyperlipidemic effect. Furthermore, the nanoparticles, nanoemulsions, and nanocapsules displayed a curative effect on hypertension and pulmonary arterial hypertension.

The delivery of phytochemicals by utilizing nanotechnology facilitated conventional medicines to link with modern techniques and improve their therapeutic efficacy. This review aims to gather all the *in vitro* and *in vivo* researches regarding plant-based nano-formulations on metabolic disorders.
